# Expression of suppressor of cytokine signaling 3 in cerebrospinal fluid after subarachnoid hemorrhage

**DOI:** 10.1186/s12974-014-0142-2

**Published:** 2014-08-14

**Authors:** Koji Osuka, Yasuo Watanabe, Masahiro Aoyama, Takahiro Nakura, Naoki Matsuo, Masakazu Takayasu

**Affiliations:** Department of Neurological Surgery, Aichi Medical University, 1-1 Yazakokarimata, Nagakute, Aichi 480-1195 Japan; High Technology Research Center, Pharmacology, Showa Pharmaceutical University, 3-3165 Higashi-tamagawa Gakuen, Machida, Tokyo 194-8543 Japan

**Keywords:** Interleukin-6, SOCS3, Subarachnoid hemorrhage

## Abstract

**Background:**

IL-6 is a proinflammatory cytokine reported to play an important role in the induction of cerebral vasospasm after subarachnoid hemorrhage (SAH). Suppressor of cytokine signaling 3 (SOCS3) is known to act as an inhibitor of signal transduction of IL-6. However, there have been no reports on the expression of SOCS3 in cerebrospinal fluid (CSF) after SAH.

**Findings:**

The concentration of IL-6 was measured serially up until day 10, in CSF of eight patients with SAH. CSF samples obtained from patients suffering from an unruptured aneurysm were used as controls. The expression of SOCS3 in CSF was further examined by immunoprecipitation methods. Concentrations of IL-6 in CSF increased immediately after the onset of SAH and remained chronically elevated over control values. SOCS3 was significantly expressed in CSF on days 1 to 3 after SAH.

**Conclusions:**

Our findings suggest that SOCS3 regulates IL-6 signaling as an antagonist in CSF, immediately following SAH. As the expression of SOCS3 decreases after day 5, IL-6 signals might then be more easily transmitted, presumably resulting in cerebral vasospasm.

**Electronic supplementary material:**

The online version of this article (doi:10.1186/s12974-014-0142-2) contains supplementary material, which is available to authorized users.

## Findings

### Introduction

Intracisternal injection of beads alone without hemorrhage in the subarachnoid space induces persistent and severe cerebral vasospasm [1], which suggests that inflammation plays an important role in the induction of cerebral vasospasm. Mathiesen *et al*. [2] first described increased IL-6 levels in cerebrospinal fluid (CSF) after subarachnoid hemorrhage (SAH), suggesting that the severe inflammatory response affects the central nervous system. In the case of severe meningitis and head trauma, severe cerebral vasospasm may be lethal. In such cases, massive production of IL-6 in CSF has been reported [3,4].

IL-6 is known to induce activation of the janus kinase/signal transducer and activator of transcription (JAK/STAT) signaling pathway and transduce signals from the cell surface into the nucleus, resulting in the transcription of immediate early genes [5]. The transcription of the suppressor of cytokine signaling 3 (SOCS3) gene increases rapidly in response to activation of IL-6, and SOCS3 acts as an inhibitor of signal transduction of IL-6 [6]. Whether the increase of IL-6 concentration in CSF after SAH is accompanied by an augmentation of SOCS3 remains unknown.

The present study was therefore undertaken to clarify the time-course of expression of SOCS3 in CSF after SAH. Our study demonstrates that SOCS3 is significantly expressed in CSF on days 1 to 3 after onset of SAH.

## Methods

### Patients and control subjects

Eight patients who underwent surgical obliteration of cerebral aneurysms at Aichi Medical University Hospital within one day after onset of SAH were enrolled in this study. The patients ranged in age from 41 to 66 years (mean 53 years); 3 were men and 5 were women. Initial clinical status was classified according to Hunt and Kosnik grade; four patients were grade II and four were grade III. The amount of blood in the subarachnoid space was defined by Fisher’s criteria; six patients were group 3 and two were group 4. Locations of all aneurysms were in the anterior circulation. After aneurysms were clipped, a cisternal catheter was inserted into the chiasmatic or prepontine cistern and postoperative CSF samples were collected serially with a drainage tube over the following ten days. Cerebral angiography or magnetic resonance angiography revealed focal cerebral vasospasm in some cases. No patients suffered from clinically symptomatic vasospasm or developed infection. Clinical outcomes were assed using the Glasgow Outcome Scale; six patients were classified as good recovery and two classified as moderate disability due to initial brain damages. As a control, CSF samples were obtained from six patients (mean 59 years) undergoing neck clipping for unruptured cerebral aneurysm. All samples were immediately centrifuged and supernatant fluids were stored at −80°C until analysis. The Ethics Committee of Aichi Medical University approved this clinical experiment.

### Analysis of IL-6 in CSF

IL-6 was measured using an enzyme immuno-assay (EIA; Catalogue Number D6050, R&D Systems, Inc., Minneapolis, MN, USA). The limit of detection of this assay was 0.7 pg/ml.

### Detection of SOCS3 in CSF

CSF samples after SAH in five patients were used for further study. CSF (500 μl) and SOCS3 polyclonal antibodies (2 μl; Catalogue Number sc-9023, Santa Cruz Biotechnology, Dallas, TX, USA) were incubated with gentle shaking for 1 hour at 4°C. After this procedure, protein G Sepharose (20 μl, GE Healthcare, Buckinghamshire, UK) was added and incubated overnight at 4°C. The immunocomplexes were then washed with PBS and boiled in 50 μl of Laemmli sample buffer for 10 minutes.

All sample buffers were centrifuged and supernatant samples were subjected to 10 % sodium dodecyl sulfate-polyacrylamide gel electrophoresis. Proteins were then transferred to polyvinylidene difluoride (PVDF) membranes under wet conditions and incubated with primary monoclonal antibodies against SOCS3 (Catalogue Number 10141, Immuno-Biological Laboratories, IBL, Gunma, Japan) at a dilution of 1:1,000 overnight at 4°C. After washing, the membranes were incubated with secondary antibodies conjugated to horseradish peroxidase (SIGMA, Saint Louis, MO, USA) at a dilution of 1:3,000 for 30 minutes at room temperature. Reactions were developed with electrochemiluminescence (ECL) (GE Healthcare). Bands were scanned with image analyzer LAS-1000 (GE Healthcare) and intensities were quantitated using the NIH IMAGE program.

### Statistical analysis

Data are represented as box-and-whisker plots indicating the median, the upper and lower quartile, the largest and the lowest value in the data set. Data are expressed as the mean ± SD values. Significant differences between the groups were assessed by one-way analysis of variance (ANOVA) followed by the Bonferroni/Dunn for multiple comparisons. Statistical significance was concluded at the *P* < 0.05 level.

## Results

### Change in IL-6 after SAH

**Figure 1:**
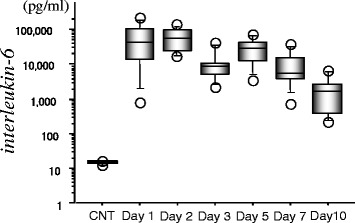
**Serial changes of IL-6 in cerebrospinal fluid (CSF) from eight patients with subarachnoid hemorrhage**. IL-6 was measured using an enzyme immuno-assay kit. Data are represented as box-and-whisker plots indicating the median, the upper and lower quartile, the largest and the lowest value. CNT, control CSF from six patients with unruptured cerebral aneurysm

CSF concentrations of IL-6 were remarkably high in the acute stage (29,869.6 ± 26,715.3 pg/ml on day 1) - more than one thousand times the control CSF value (11.4 ± 0.9 pg/ml) -and remained chronically elevated (Figure 1).

### Expression of SOCS3 after SAH

**Figure 2:**
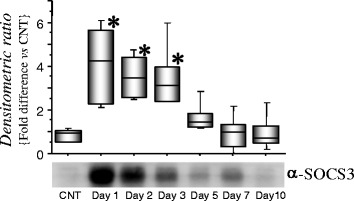
**Expression of suppressor of cytokine signaling 3 (SOCS3) in cerebrospinal fluid (CSF) from five patients**. SOCS3 was immunoprecipitated from CSF with polyclonal anti-SOCS3 antibodies, and the resulting immunocomplexes were subjected to immunoblot analysis using monoclonal anti-SOCS3 antibodies. Data show the amount of SOCS3 after subarachnoid hemorrhage (SAH) relative to that of the control, using the values of the median, the upper and lower quartile with maximum/minimum whiskers. Representative western blot analysis from one individual is shown (days 1 to 10). CNT, control CSF from unruptured cerebral aneurysm; *P < 0.05 denotes a significant difference between the control and SAH groups, determined by analysis of variance followed by the Bonferroni/Dunn for multiple comparisons.

SOCS3 has been previously identified as an inhibitor of the JAK/STAT signaling pathway activated by IL-6. We examined the expression of SOCS3 in the CSF by immunoprecipitation methods. As shown in Figure 2, SOCS3 was significantly expressed on days 1 to 3 after onset of SAH compared with controls and days 5 to 7 post-SAH.

## Discussion

In this study we demonstrated that concentrations of IL-6 in CSF increase immediately after the onset of SAH and are maintained for a week after the event. In addition, a significant increase in SOCS3 in CSF was confirmed on days 1 to 3 by immunoprecipitation.

Significant increases in IL-6 levels in CSF after SAH have been previously reported [2,7-9], which is consistent with our data. Recently, Schoch *et al*. argued that IL-6 is a reliable early marker for predicting vasospasm on days 4 and 5 before clinical onset [9]. Intracisternal injection of IL-6 itself induces long-lasting vasoconstriction in canines [8]. Moreover, IL-6 is known to induce phosphorylation of JAK1 and STAT3 in the rat basilar artery after SAH and transduces signals into the nucleus [10], resulting in the transcription of immediate early genes. Considering these data, IL-6 might play an important role in inducing cerebral vasospasm through the regulation of this JAK/STAT signaling pathway.

The SOCS protein is one of the key factors involved in the negative regulation of JAK/STAT signaling [6]. SOCS genes are transcriptionally activated in response to STAT-mediated mechanisms. SOCS proteins interact with the catalytic domains of JAK proteins and inhibit JAK kinase activity, resulting in the inhibition of STAT activation [11]. This negative feedback pathway tightly regulates cytokine-induced activation of STATs. IL-6 promotes acute and chronic inflammatory disease in SOCS3 knock-out mice *in vivo* [12]. In response to vascular injury, the JAK/STAT signaling pathway is involved with neointimal formation and atherosclerotic plaque progression [13,14]. Taken all together, SOCS3 might regulate the JAK/STAT signaling pathway activated by IL-6, and subsequent transcription of genes in the basilar artery immediately after SAH. Although no apparent relationship between outcome, IL-6 levels and SOCS3 was obtained from this limited study [see Additional file 1: Figure S1], we speculate that the expression of SOCS3 decreases after day 5 and IL-6 signals might then be more easily transmitted, presumably resulting in cerebral vasospasm. Cerebral vasospasm is a relevant problem that affects outcome [15,16]. The use of SOCS3 could be one of the modalities to reduce cerebral vasospasm in the future.

## Conclusion

The present investigation has, for the first time, clarified chronological expressions of SOCS3 in CSF after SAH. Of particular note is our finding that SOCS3 is significantly expressed in CSF on days 1 to 3 after onset of SAH, suggesting that SOCS3 may inhibit IL-6 signal transduction immediately after SAH. Further studies using SOCS3 as an antagonist of IL-6 should be conducted to more precisely clarify the mechanisms of IL-6 regulation after SAH.

### Ethics approval

The study was conducted with the approval of the Aichi Medical University Ethics Committee.

## Additional file

Additional file 1: Figure S1. Relationship of IL-6 and suppressor of cytokine signaling 3 (SOCS3) in cerebrospinal fluid (CSF) from five patients. We measured the concentrations of IL-6 within CSF on day 1 from five more patients. Three CSF samples contained IL-6 less than 10,000 pg/ml (low group) and 2 CSF samples contained IL-6 more than 20,000 pg/ml (high group). SOCS3 was immunoprecipitated with polyclonal anti-SOCS3 antibodies from these five CSFs, and the resulting immunocomplexes were subjected to immunoblot analysis using monoclonal anti-SOCS3 antibodies. Western blot analysis revealed that band intensity of SOCS3 was detected more clearly from the high group than the low group, suggesting that there might be a tight relationship between IL-6 and SOCS3.

## Abbreviations

ANOVA: analysis of variance
CSF: cerebrospinal fluid
ECL: electrochemiluminescence
IL: interleukin
JAK: janus kinase
PVDF: polyvinylidene difluoride
SAH: subarachnoid hemorrhage
SOCS3: suppressor of cytokine signaling 3; STAT:signal transducer and activator of transcription
