# Supplementary figures and images for: Expression of suppressor of cytokine signaling 3 in cerebrospinal fluid after subarachnoid hemorrhage
Source: J Neuroinflammation. 2014 Aug 14;11:142. doi: 10.1186/s12974-014-0142-2 (PMC4243948; doi:10.1186/s12974-014-0142-2)

## Slide 1
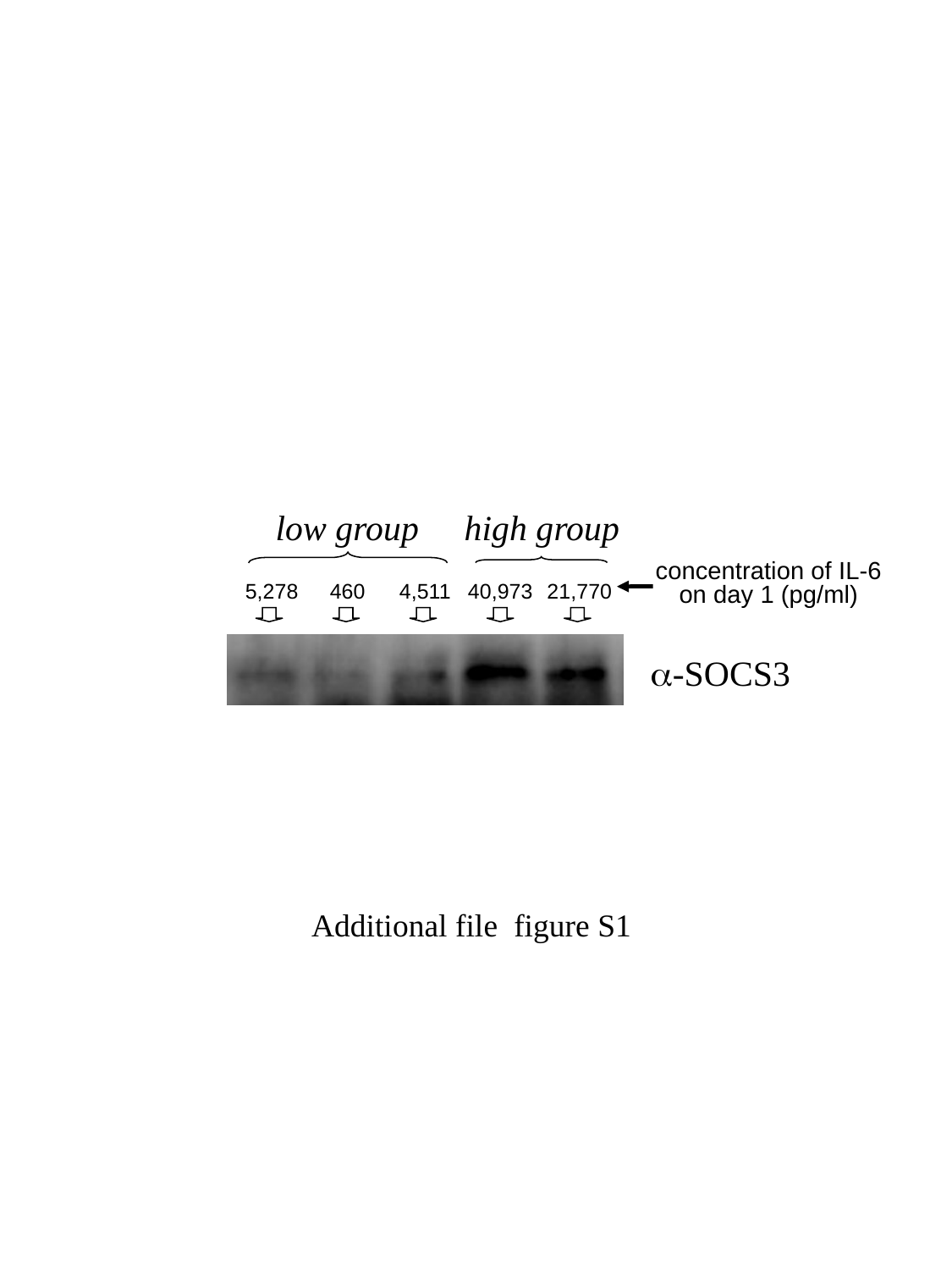

low group
high group
concentration of IL-6
on day 1 (pg/ml)
5,278
460
4,511
40,973
21,770
α-SOCS3
Additional file figure S1

Supplement: Additional file 1: Figure S1. — Relationship of IL-6 and suppressor of cytokine signaling 3 (SOCS3) in cerebrospinal fluid (CSF) from five patients. We measured the concentrations of IL-6 within CSF on day 1 from five more patients. Three CSF samples contained IL-6 less than 10,000 pg/ml (low group) and 2 CSF samples contained IL-6 more than 20,000 pg/ml (high group). SOCS3 was immunoprecipitated with polyclonal anti-SOCS3 antibodies from these five CSFs, and the resulting immunocomplexes were subjected to immunoblot analysis using monoclonal anti-SOCS3 antibodies. Western blot analysis revealed that band intensity of SOCS3 was detected more clearly from the high group than the low group, suggesting that there might be a tight relationship between IL-6 and SOCS3. [file 12974_2014_142_MOESM1_ESM.ppt]
